# Neuromedin U Receptor NMUR3 Regulates Autophagy, Thereby Enhancing Thermal Tolerance in *C. elegans*

**DOI:** 10.3390/ijms26178471

**Published:** 2025-08-31

**Authors:** Limei He, Jianqi Yang, Shufang Wang, Yicheng Ma, Chenggang Zou

**Affiliations:** 1 State Key Laboratory for Conservation and Utilization of Bio-Resources in Yunnan and Key Laboratory of Industrial Microbial Fermentation Engineering of Yunnan Province, School of Life Sciences, Yunnan University, Kunming 650091, China; hlm2025666@163.com (L.H.); wangsf202508@163.com (S.W.); m410610@163.com (Y.M.); 2 Faculty of Health Sciences, University of Macau, Macau 999078, China; cc21798@um.edu.mo

**Keywords:** NMURS, *Caenorhabditis elegans*, NMUR-3, heat shock, AMPK, autophagy

## Abstract

Neuromedin U receptors (NMURs) represent a class of evolutionarily conserved G-protein-coupled receptors that play a pivotal role in a variety of physiological processes. However, the role of NMURs in the heat shock response has yet to be elucidated. Using the nematode *Caenorhabditis elegans* as a model system, we demonstrate herein that functional loss of NMUR-3 results in reduced survival upon heat shock. The regulation of thermal tolerance by NMUR-3 is dependent on AMPK. Furthermore, our data demonstrate that NMUR-3 activates autophagy via AMPK, thereby contributing to protection against heat shock. The results of this study suggest that NMUR-3 is crucial for thermal tolerance in *C. elegans*.

## 1. Introduction

The nematode *Caenorhabditis elegans* has evolved various strategies to adapt to different stresses, both biotic and abiotic. It is widely recognized that the SKN-1/Nrf2 pathway has been shown to activate antioxidant defenses [1,2], with p38 MAPK signaling being implicated in the mediation of pathogen resistance [3,4]. Furthermore, SKN-1/Nrf2 has been demonstrated to directly stimulate the expression of glutathione transferase (GST-4) [5,6]; in comparison, DAF-16/FOXO has been shown to increase superoxide dismutase (SOD) activity, thereby mitigating oxidative damage [7]. In response to osmotic stress, *C. elegans* exhibits a regulatory response involving the modulation of ion channels, such as sodium/hydrogen exchangers [8]. The authors of several studies have highlighted the role of autophagy in enhancing biotic and abiotic resistance in *C. elegans* [9,10].

The transcription factor heat shock factor 1 (HSF-1) plays a central role in heat stress response (HSR) by activating the expression of a constellation of heat shock proteins (HSPs) [11]. Elevated temperature activates the HSR, in which HSF-1 induces the expression of HSP-70 and HSP-16 to restore protein homeostasis by refolding misfolded proteins in worms [12]. In addition to the process of transcriptional reprogramming of the HSR, the ability of *C. elegans* to respond to heat shock is influenced by several other factors. For instance, inhibition of the insulin/IGF-1 signaling (IIS) pathway enhances heat resistance via the downstream transcription factor DAF-16/FOXO, which regulates stress-responsive genes [13]. In addition, the epigenetic regulators CBP-1 and SWSN-1 play a role in regulating longevity in *C. elegans* following early heat stress [14,15].

Neuropeptides and their receptors, as the key mediators of intercellular communication, are widely involved in regulating metabolism, behavior, and stress responses. As a highly conserved neuropeptide originally identified in the porcine spinal cord in mammals, neuromedin U (NMU) is so named because of its potent uterine-contraction-inducing activity [16]. NMU can transmit its signals through the NMU receptor (NMUR) family, which are types of G protein-coupled receptors (GPCRs) [17]. These receptors consist of different tissue distributions, enabling NMU to produce distinct physiological effects in different organs [18]. In the *C. elegans* genome, three orthologs of the NMUR family exist, named NMUR-1 to NMUR-3 [19]. The authors of recent studies on the NMU receptor family in *C. elegans* primarily focus on NMUR-1 [20,21,22,23]. NMUR-1 acts with the sensory system to affect the lifespan of worms in a manner dependent on the bacterial lipopolysaccharide structure [20]. Moreover, the NMUR-1 signaling pathway has been shown to be essential for diet-induced changes in mitochondrial function and the process of premature aging [23]. In addition, NMUR-1 is essential for the retrieval of learned salt avoidance, in addition to the regulation of distinct immune responses to different pathogens [21,22]. At present, the primary function of NMUR-2 and NMUR-3 remains unknown.

In this study, we aim to explore the role of NMUR-3 in a range of stressors, including biotic stresses (*Pseudomonas aeruginosa* PA14) and abiotic stresses (high salt, heat shock, and senescence). Our data demonstrated that the functional loss of NMUR-3 significantly reduced survival under heat shock in *C. elegans*. Through genetic screening, we found that thermal tolerance in worms regulated by NMUR-3 was dependent on AMPK-mediated autophagy. Thus, NMUR-3 plays a key role in thermal tolerance in *C. elegans*.

## 2. Results

### 2.1. NMUR-3 Mediates Resistance to Heat Shock in Worms

**Figure 1:**
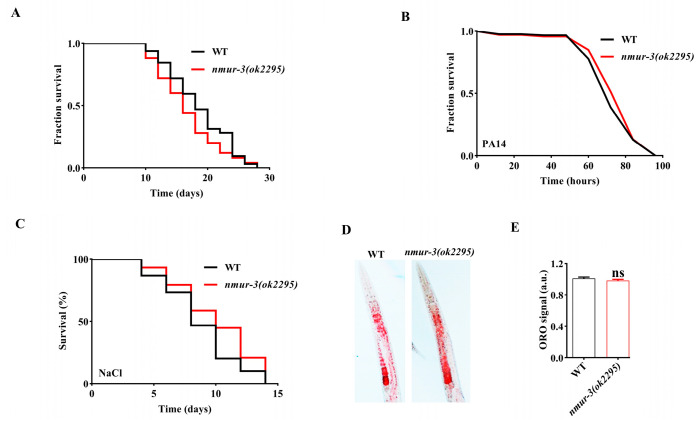
*nmur-3* is not involved in the regulation of lifespan, resistance to *P. aeruginosa* PA14, high salt tolerance, and lipid metabolism in *C. elegans*. (**A**) The lifespan assay of *nmur-3 (ok2295)* mutants and wild-type (WT) worms exhibited no significant difference. (**B**) No significant difference was found in the survival of *nmur-3 (ok2295)* mutants and WT worms when *exposed* to *P. aeruginosa* PA14. (**C**) No significant difference was found in the survival of *nmur-3 (ok2295)* mutants and WT worms when they were exposed to 300 mM NaCl. *p*-values (**A**–**C**) were calculated using a log-rank test. (Shown in Supplementary Table S1). (**D**) Oil Red O staining of *nmur-3 (ok2295)* mutants and WT worms grown on *E. coli* OP50. (**E**) The quantification of Oil Red O staining revealed comparable fat levels in *nmur-3 (ok2295)* mutants and WT worms. ns, not significant. The results show the means ± SD of three independent experiments. The *p*-value was calculated using Student’s t-test.

**Figure 2:**
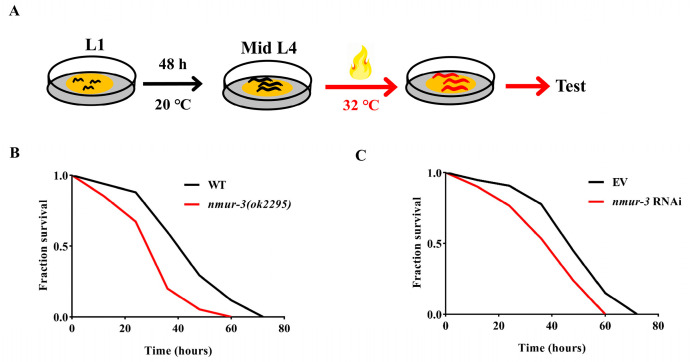
The ability of worms to withstand high temperatures depends on *nmur-3*. (**A**) Schematic illustration of test conditions. (**B**) The survival rate of *nmur-3 (ok2295)* mutants was lower than that of WT worms when they were exposed to 32 °C. (**C**) The survival rate of worms subjected to *nmur-3* RNAi was lower than that of worms subjected to an empty vector (EV) when they were exposed to 32 °C. *p*-values (**B**,**C**) were calculated using a log-rank test (shown in Supplementary Table S1).

In order to ascertain the potential impact of *nmur-3* on the lifespan of *C. elegans*, lifespan measurements were conducted on the *nmur-3 (ok2295)* mutants. The results revealed no differences in lifespan between the *nmur-3 (ok2295)* mutants and the wild-type (WT) worms (Figure 1A). Compared with WT worms, *nmur-3 (ok2295)* mutants exhibited no differences in survival against *P. aeruginosa* PA14 and high-salt conditions (NaCl 300 mM) (Figure 1B,C). In addition, the quantity of lipid droplets in the intestine, determined based on Oil Red O staining, showed no significant difference between the *nmur-3 (ok2295)* mutants and the WT worms (Figure 1D,E). Furthermore, under heat shock conditions (Figure 2A), *nmur-3 (ok2295)* mutants displayed markedly lower survival than that of WT worms (Figure 2B). This finding was also confirmed in *nmur-3* RNAi worms (Figure 2C). The above results therefore suggest that NMUR-3 is crucial for heat shock responses in *C. elegans*.

### 2.2. NMUR-3-Mediated Thermal Tolerance Is Dependent on AMPK in Worms

**Figure 3:**
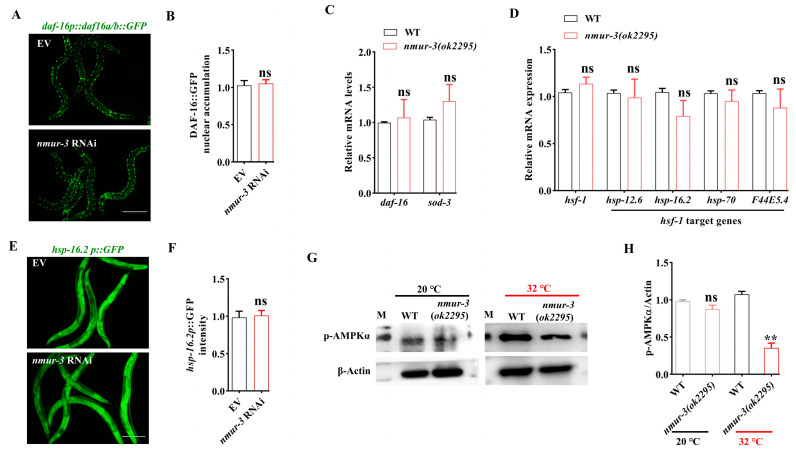
The phosphorylation of AMPK under heat shock is regulated by *nmur-3* in *C. elegans*. (**A**) Representative images of the *daf-16p::daf-16a/b::GFP* transgenic worms. Scale bar: 100 μm. (**B**) Quantification of DAF-16::GFP revealed that knockdown of *nmur-3* did not impact the nuclear translocation of DAF-16 in worms exposed to 32 °C. ns, not significant. The results are presented as the means ± SD of three independent experiments. (**C**) Quantitative RT-qPCR analysis revealed no alteration in *daf-16* or its transcriptional target *sod-3* expression in heat-shocked (32 °C) *nmur-3 (ok2295)* mutants relative to WT. ns, not significant. The results are presented as the means ± SD of three independent experiments. (**D**) Quantitative RT-qPCR analysis detected unaltered mRNA levels of *hsf-1* and its transcriptional targets (*hsp-12.6*, *hsp-16.2*, *hsp-70*, *F44E5.4*) in *nmur-3 (ok2295)* mutants following 32 °C heat shock relative to WT. ns, not significant. The results are presented as the means ± SD of three independent experiments. (**E**) Representative images of the *hsp-16.2p::gfp* transgenic worms. Scale bar: 100 μm. (**F**) Quantification of *hsp-16.2p::gfp* revealed that knockdown of *nmur-3* did not affect the expression of *hsp-16.2* in worms exposed to 32 °C. ns, not significant. The results are presented as the means ± SD of three independent experiments. (**G**) Representative images of the Western blot detection of AMPKα phosphorylation. M, marker. (**H**) The phosphorylation levels of AMPKα were reduced in *nmur-3 (ok2295)* mutants compared to WT worms when they were exposed to 32 °C. ** *p* < 0.01. ns, not significant. The results are presented as the means ± SD of three independent experiments. *p*-values (**B**–**D**,**F**,**H**) were calculated using Student’s *t*-test.

In order to elucidate the molecular basis of *nmur-3* mediated thermal tolerance, we examined the activation of DAF-16, which increases nuclear translocation in response to heat shock [24]. We found that heat shock-induced nuclear localization of DAF-16 did not impact in *nmur-3* RNAi worms (Figure 3A,B). Furthermore, we found that RT-qPCR analysis of heat-shocked *nmur-3 (ok2295)* mutant worms revealed no significant alterations in the mRNA levels of *daf-16* or its target gene *sod-3* [25] (Figure 3C). Additionally, we assessed the mRNA levels of the heat shock transcription factor *hsf-1* and its target genes (*hsp-12.6*, *hsp-16.2*, *hsp-70*, and *F44E5.4*) [26], which also remained unaltered in *nmur-3 (ok2295)* mutants after heat shock (Figure 3D). A widely used marker for heat shock response is the heat shock inducible gene *hsp-16.2*, a target gene of the heat shock transcription factor HSF-1 [27,28]. Using the transgenic worms expressing *hsp-16.2p::gfp*, we found that knockdown of *nmur-3* by RNAi did not impact the expression of *hsp-16.2* in worms after heat shock (Figure 3E,F). These data suggest that activation of DAF-16 and HSF-1 is not regulated by NMUR-3 in worms upon heat shock.

**Figure 4:**
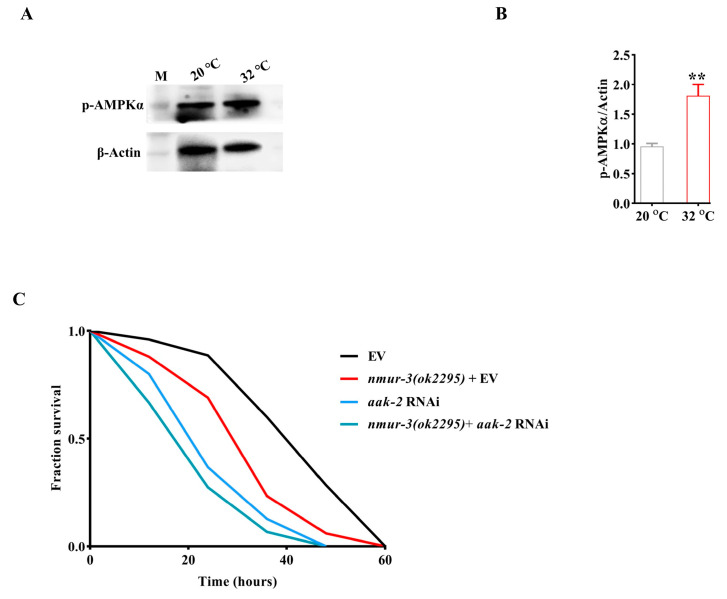
The regulation of thermal tolerance in worms by *nmur-3* is dependent on *aak-2*. (**A**) Representative images of the Western blot detection of AMPKα phosphorylation. M, marker. (**B**) The levels of AMPKα phosphorylation were increased in WT worms exposed to 32 °C compared to WT worms exposed to 20 °C. ** *p* < 0.01. The results are presented as the means ± SD of three independent experiments. The *p*-value was calculated using Student’s t-test. (**C**) The survival rate of *nmur-3 (ok2295)* mutants or *aak-2* RNAi worms was lower than that of EV worms when they were exposed to 32 °C. The survival rate of worms was no longer affected in *nmur-3 (ok2295) + aak-2* RNAi compared to *aak-2* RNAi worms when they were exposed to 32 °C. The *p*-value was calculated using a log-rank test (shown in Supplementary Table S1).

AMPK is also implicated in various stress responses such as heat shock [29]. We therefore speculate that the resistance of NMUR-3 to heat shock may be related to AMPK. AMPK is activated by the phosphorylation of the α-subunit by Thr172 [30]. We observed that there was not altered in the levels of phospho-AMPKα in *nmur-3 (ok2295)* mutants compared to WT worms fed with *E. coli* OP50 at a temperature of 20 °C (Figure 3G,H). Moreover, the protein levels of AMPK phosphorylation were reduced in *nmur-3 (ok2295)* mutants compared to WT worms after heat shock (Figure 3G,H). Importantly, the levels of AMPK phosphorylation significantly increased in WT worms after heat shock (Figure 4A,B). Meanwhile, we found that there was not altered in the mRNA levels of the catalytic subunit gene *aak-1* and regulatory subunit gene *aak-2* in WT worms after heat shock (Figure S1). The mRNA levels of *aak-1* and *aak-2* remained unchanged in *nmur-3 (ok2295)* mutants after heat shock or at a temperature of 20 °C (Figure S1). These data indicate that *nmur-3* promotes AMPK activation during heat shock. Moreover, we found that *aak-2* RNAi decreased survival to a significantly lesser extent than *nmur-3 (ok2295)* mutants after heat shock (Figure 4C). However, the *nmur-3 (ok2295)* mutants subjected to *aak-2* RNAi did not exhibit further reduction in survival compared to *nmur-3 (ok2295)* mutants after heat shock (Figure 4C). These results suggest that the regulation of thermal tolerance by NMUR-3 in worms is dependent on AMPK.

### 2.3. NMUR-3 Increases Heat Resistance by Inducing Autophagy via AMPK

**Figure 5:**
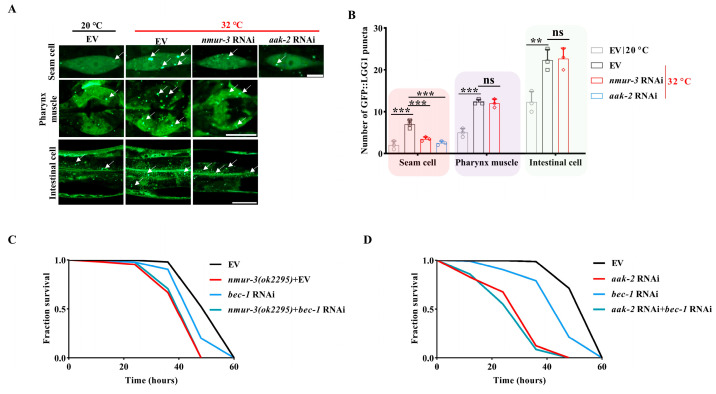
The regulation of thermal tolerance in worms by NMUR-3 is dependent on autophagy. (**A**) Representative images of GFP::LGG-1 puncta in a seam cell (scale bar: 2 μm), pharynx muscle (scale bar: 5 μm), and an intestinale cell (scale bar: 5 μm). The arrow indicates a typical GFP::LGG-1 puncta. (**B**) Quantification of the number of GFP::LGG-1 puncta in worms. The number of GFP::LGG-1 puncta in the seam cell was reduced in *nmur-3* RNAi worms or *aak-2* RNAi worms compared to EV worms when they were exposed to 32 °C. ** *p* < 0.01; *** *p* < 0.001. ns, not significant. The results are presented as the means ± SD of three independent experiments. The *p*-value was calculated using a one-way ANOVA followed by a Student–Newman–Keuls test. (**C**) The survival rate of *nmur-3 (ok2295)* mutants or *bec-1* RNAi worms was lower than that of EV worms when they were exposed to 32 °C. The survival rate of worms was no longer affected in *nmur-3 (ok2295)* + *bec-1* RNAi compared to *bec-1* RNAi worms when they were exposed to 32 °C. (**D**) The survival rate of *bec-1* RNAi worms or *aak-2* RNAi worms was lower than that of EV worms when they were exposed to 32 °C. The survival rate of worms was no longer affected in *aak-2*+*bec-1* double RNAi worms compared to *aak-2* RNAi worms when they were exposed to 32 °C. *p*-values (**C**,**D**) were calculated using a log-rank test (shown in Supplementary Table S1).

Autophagy is essential for heat survival in *C. elegans* [31]. We investigated whether *nmur-3* mediates the heat resistance of worms by promoting autophagy. Consistent with the findings of a previous study [31], we detected an increase in GFP::LGG-1/Atg8 puncta in the hypodermal seam cell, pharynx muscle, and intestinal cell of worms subjected to heat shock (Figure 5A,B). However, knockdown of *nmur-3* by RNAi markedly reduced the number of GFP::LGG-1 puncta in the hypodermal seam cell of worms after heat shock (Figure 5A,B). No changes in the pharynx muscle or intestinal cell were observed (Figure 5A,B). In addition, knockdown of *nmur-3* by RNAi did not impact the number of GFP::LGG-1 puncta in the hypodermal seam cells of WT worms fed with *E. coli* OP50 at a temperature of 20 °C (Figure S2A,B). Furthermore, the number of GFP::LGG-1 puncta was significantly lower in the hypodermal seam cells of worms subjected to *aak-2* RNAi after heat shock (Figure 5A,B).

To further investigate whether NMUR-3/AMPK signaling mediates heat resistance by upregulating autophagy, the *bec-1*/ATG6 autophagy gene was knocked down in *nmur-3 (ok2295)* mutants and *aak-2* RNAi worms, respectively. We found that *bec-1* RNAi decreased survival to a significantly lesser extent than *nmur-3 (ok2295)* mutants or *aak-2* RNAi worms after heat shock (Figure 5C,D). However, the *nmur-3 (ok2295)* mutants subjected to *bec-1* RNAi did not exhibit a further reduction in survival compared to *nmur-3 (ok2295)* mutants after heat shock (Figure 5C). In addition, the *aak-2+bec-1* double RNAi did not further reduce survival compared to *aak-2* RNAi worms after heat shock (Figure 5D). Collectively, these results suggest that NMUR-3 enhances the heat resistance of worms by activating AMPK-mediated autophagy.

## 3. Discussion

In this study, we investigated the role of the *C. elegans* NMU receptor, NMUR-3, in resistance to pathogens, tolerance to high salt and temperature, aging, and lipid metabolism. Our findings demonstrated that NMUR-3 regulates autophagy by modulating AMPK activation, leading to increased resilience to high temperatures. The results of previous studies have demonstrated that NMUR-1 is involved in regulating aging, salt avoidance, and innate immune response in *C. elegans* [20,21,22,23]. In the present study, *nmur-3* mutants exhibited no survival differences after *P. aeruginosa* PA14 infection. Furthermore, NMUR-1 regulates the lifespan of *C. elegans* through HSF-1 and DAF-16 [20]. However, our data indicate that NMUR-3 is dispensable for longevity. Nevertheless, the regulation of thermal tolerance by NMUR-3 in worms is due to the activation of AMPK, but not HSF-1 and DAF-16. These findings suggest that the NMUR family is functionally differentiated in *C. elegans*, with resistance to heat shock largely dependent on NMUR-3. In *C. elegans*, CAPA-1 has been demonstrated to be a homolog of vertebrate NMU and insect capability peptides, acting as a ligand of the NMUR-1 receptor. However, the ligand of either the NMUR-2 or NMUR-3 receptor remains to be identified in worms.

Activation of the AMPK-SIRT1-PGC-1α pathway increases the expression of HSP-70, thus reducing heat stress-induced fat deposition in porcine subcutaneous preadipocytes [32]. In addition, heat stress triggers protective autophagy via the ATP-AMPK-mTOR pathway, thereby preventing heat-induced apoptosis in hepatocellular carcinoma [33]. However, the results of a previous study indicate that heat stress in fact inhibits the AMPK-PGC-1α signaling pathway by reducing its expression in 3T3-L1 preadipocytes [34]. At present, the role of AMPK in heat shock remains unclear in *C. elegans*. Our findings demonstrate that heat stress increases phospho-AMPKα levels in *C. elegans*. Notably, knockdown of *aak-2* by RNAi leads to increased susceptibility of worms after heat shock. The results of a previous study by Kumsta et al. indicate that the activation of autophagy via heat shock is required for thermal tolerance in *C. elegans* [31]. However, the exact mechanism by which heat stress activates autophagy remains unknown. The results of the present study demonstrate that AMPK induces autophagy via heat stress in *C. elegans*. AMPK therefore functions as a downstream molecule of NMUR-3 to mediate thermotolerance by activating autophagy. It should be noted that, although heat shock induces autophagy throughout the entire body of worms, NMUR-3 only mediates the autophagy activation in seam cells. It is evident that the mechanism of heat stress-mediated autophagy requires further investigation in light of our current results.

Our findings suggest a conserved evolutionary mechanism in which the energy sensor AMPK integrates multiple stress signals through autophagy regulation, with *nmur-3* potentially serving as a thermal stress signal that initiates adaptive responses. Nevertheless, the discovery of the core role of the NMUR-3–AMPK–autophagy axis in heat shock tolerance provides a novel molecular framework for the development of NMUR-targeted neuroprotective agents. The results of this study provide a novel approach that enhances our understanding of the mechanisms underlying NMUR-mediated stress resistance in *C. elegans*, providing insights into heat stress tolerance mechanisms.

## 4. Materials and Methods

### 4.1. Nematode Strains and Bacteria

N2 Bristol was used as the wild-type strain in the present study. The transgenic strains used, including VC1974 (*F02E8.2(ok2295)*), DA2123 (adIs2122 [*lgg-1p::GFP::lgg-1* + *rol-6(su1006)*]), TJ375 (*gpIs1 [hsp-16.2p::GFP]*), and TJ356 (*zIs356 [daf-16p::daf-16a/b::GFP + rol-6(su1006)]*) were kindly provided by the Caenorhabditis Genetics Center (CGC; http://www.cbs.umn.edu/CGC accessed on 1 June 2022), funded by the NIH Office of Research Infrastructure Programs (P40 OD010440). All strains were maintained on nematode growth media (NGM) and fed with *E. coli* OP50 at 20 °C [35].

### 4.2. Synchronization Methods

Egg-bearing *C. elegans* were harvested in M9 buffer into 1.5 mL microcentrifuge tubes. Worms were pelleted by brief centrifugation (room temperature; repeated 2–3 times) to remove surface bacterial contamination; natural sedimentation was an alternative. The M9 supernatant was removed, and worms were lysed in 1 mL lysis buffer via vigorous vortexing until granular. Lysis was terminated by immediate brief centrifugation and supernatant removal. Pellets were washed twice with M9 buffer to remove residual sodium hypochlorite. Eggs were resuspended in 1 mL M9 buffer, transferred to culture dishes containing M9 buffer, and incubated at 20 °C for development.

### 4.3. Oil Red O (ORO) Staining

ORO staining of lipids was performed following a previously described protocol with minor adjustments [36]. Briefly, the young adult worms were collected and washed with 1× PBS buffer (Sangon Biotech, Shanghai, China, E607008) until clear. Thereafter, 250 µL of 1× PBS buffer, 125 µL of 2× modified Ruvkun’s witches brew (MRWB) solution, and 4% paraformaldehyde were added to the suspension. Following fixation, the sample underwent two freeze–thaw cycles in liquid nitrogen. The samples were then washed three times with 1× PBS, and the supernatant was discarded, with 100 µL being retained. Next, 100 µL of 60% isopropanol was added and mixed at room temperature for 5 min, followed by isopropanol removal. Thereafter, 1 mL of ORO staining solution (Servicebio, Wuhan, China, G1015) was added and incubated in the dark at room temperature on a shaker (Tensuc, Shanghai, China, TS-300DC) at 100 rpm for 10–14 h. The sample was washed with 1× PBS until no red color remained. Slides were observed under an Olympus color camera (Olympus Corporation, Tokyo, Japan) outfitted with DIC optics, and images were captured. Anterior intestinal cell areas were selected to determine Oil Red O intensities. Fifteen worms were examined per assay in three independent experiments. ORO staining was quantified using ImageJ (v1.53, NIH) software by measuring the intensity in an equal-sized intestinal area in each image.

### 4.4. RNAi in C. elegans

RNAi studies in the worms were performed by using the standard feeding method and the RNAi bacterial strains were obtained from the Ahringer RNAi library [37]. To conduct RNA interference experiments on the worms, the following feeding method was used: interference strains were obtained from the *C. elegans* RNAi library, streaked onto ampicillin-resistant LB plates, and incubated overnight at 37 °C. A single colony was selected and inoculated into ampicillin-resistant LB liquid. The culture was incubated overnight at 37 °C in a shaking incubator (Tensuc, Shanghai, China, TS-300DC), and the sequences were verified to ensure accuracy. The bacterial culture was centrifuged and concentrated, and a portion of the concentrated bacterial solution was added to IPTG (5 mM) induction plates containing ampicillin (100 μg/mL). The plates were induced for 18–24 h to ensure dsRNA production at 25 °C. L1 larvae were placed on the seeded RNAi plates at 20 °C until the animals reached the mid–L4 stage.

### 4.5. Lifespan Analysis

Young adult worms were rinsed with M9 buffer and collected. They were then placed on FUDR-NGM plates, incubated at 20 °C, and transferred to fresh plates every alternate day. Survival was monitored until all worms had died; a worm was considered dead if it did not respond to three sequential taps on the head and tail. Each lifespan experiment was conducted three times independently, with 50–60 worms per experiment. Information on the sample sizes used for each assay is provided in Table S1.

### 4.6. C. elegans Infection Experiment

In this study, infection experiments were performed primarily according to method [38]. Briefly, the PA14 bacteria were incubated overnight at 37 °C in a shaking incubator (Tensuc, Shanghai, China, TS-300DC). An appropriate volume of the bacterial solution was pipetted and spread evenly onto FUDR-NGM plates. These plates were incubated at 37 °C for 12 h, with the temperature then decreased to 25 °C for an additional 12 h. Mid–L4 worms were added to these plates, ensuring that the total number did not exceed 90 worms. Survival rates were monitored and recorded every 12 h until all worms were dead; a worm was considered dead if it did not respond to three sequential taps on the head and tail. Information on the sample sizes used for each assay is provided in Table S1.

### 4.7. C. elegans Stress Response Experiment Under Heat Shock and High-Salt Conditions

In this study, infection experiments were conducted primarily based on method [39], with minor modifications. Briefly, synchronized L1 worms were grown on NGM or NGM–IPTG plates until they reached the young adult stage at 20 °C. The worms were then transferred to fresh OP50 NGM plates at 32 °C for the heat shock experiment. Following a two-hour period of heat shock, a series of assays were conducted, encompassing the measurement of GFP fluorescence (*hsp-16.2p::GFP*, and *daf-16p::daf-16a/b::GFP*), the observation of autophagy (*lgg-1p::GFP::lgg-1*), RT-qPCR and Western blot analysis. To provide high-salt conditions, the worms were moved to NGM plates containing 300 mM NaCl. Survival rates were monitored and recorded every 12 h until all worms died; a worm was considered dead if it did not respond to three sequential taps on the head and tail. Three plates of fifty to sixty worms per plate were tested per assay, and all experiments were performed three times independently. Information on the sample sizes used for each assay is provided in Table S1.

### 4.8. RNA Extraction, cDNA Synthesis and RT-qPCR Analysis

Total RNA was isolated from *C. elegans* (washed with M9 buffer) using TRIzol reagent (Invitrogen, Carlsbad, CA, USA). Worms underwent seven liquid nitrogen freeze–thaw cycles (35 °C incubation shaker), followed by homogenization in TRIzol (100 µL per 1 mL sample). Samples were centrifuged (12,000× *g*, 15 min, 4 °C), and supernatants were transferred to nuclease-free tubes. Chloroform (200 µL) was added, samples were vortexed (15 s), incubated (10 min), and centrifuged (12,000× *g*, 15 min, 4 °C). The aqueous phase was mixed with equal-volume isopropanol, incubated (10 min), and centrifuged (12,000× *g*, 15 min, 4 °C). RNA pellets were washed twice with 75% ethanol (12,000× *g*, 5 min, 4 °C), air-dried in a laminar flow hood, and dissolved in 60 µL DEPC-treated water. RNA quality was assessed by electrophoretic verification of integrity, while concentration was determined spectrophotometrically (NanoDrop 2000, Thermo Fisher Scientific, Waltham, MA, USA) before cryopreservation at −80 °C.

cDNA was synthesized from 1 µg total RNA using the *Evo M-MLV* RT Premix kit (Accurate Biology, Changsha, China, AG11728) with gDNA removal (42 °C, 2 min). RT-qPCR was performed using SYBR Green Pro Taq HS Premix (Accurate Biology, Changsha, China, AG11701) on a Roche Light Cycler 480 (Roche Diagnostics, Basel, Switzerland). Reactions (20 µL) contained 10 µL master mix, gene-specific primers (0.2 µM each), and 2 µL cDNA template. Cycling conditions: 95 °C/30 s; 40 cycles of 95 °C/5 s and 60 °C/30 s. Melt curve analysis confirmed amplification specificity. Sequences for all primer pairs used in this study can be found in Table S2. For quantification of mRNA levels following heat shock, animals were harvested immediately after the heat shock conditions specified (32 °C, 2 h). Relative quantification used the 2^(−ΔΔCt)^ method with GraphPad Prism 9.5.1 (GraphPad Software Inc., La Jolla, CA, USA) for statistical analysis.

### 4.9. Microscopy

The transgenic worms carrying *lgg-1p::GFP::lgg-1*, *hsp-16.2p::GFP*, and *daf-16p::daf-16a/b::GFP* were mounted in M9 onto microscope slides for detecting fluorescent signals. Fluorescence microscopy was conducted on a Zeiss Axioskop 2 Plus system (Zeiss, Oberkochen, Germany) equipped with FITC filters (ex 488 nm/em 509 nm) to visualize *hsp-16.2p::GFP* and *daf-16p::daf-16a/b::GFP* transgenic worms. For the transgenic worms carrying GFP::LGG-1, GFP::LGG-1 positive puncta were imaged using a Zeiss confocal microscope (LSM900, Axio Observer, Zeiss, Oberkochen, Germany) using a ×63 oil objective (Plan-Apochromat ×63/1.40 oil DIC M27), a 1 Airy Unit/45 µm pinhole (0.5 µm optical section thickness), and Zen software (3.0, blue). GFP::LGG-1 positive puncta were counted in the seam cells, intestine, and pharynx muscle. Thirty worms were examined per assay in three independent experiments. The fluorescence intensity was analyzed using ImageJ (v1.53, NIH).

### 4.10. Western Blot

Briefly, we first collected 100 μL of worms and added 1/100 PMSF and phosphatase inhibitors. Next, we sonicated the mixture at 4 °C (30 s on/30 s off) for 1 min. Centrifugation was performed at 12,000 rpm for 15 min at 4 °C. We collected the supernatant, quantified the protein concentration using the BCA assay, and then added the protein loading buffer. We subsequently boiled the samples for 10 min. Next, 50 μg of total protein lysates per sample was loaded and separated onto 12% SDS-PAGE gel. Thereafter, proteins were transferred to PVDF membranes (Millipore, Bedford, MA, USA) for antibody incubation. The primary antibodies included anti-Phospho-AMPKα (Thr172) antibodies (2535, 1:1000 dilution, Cell Signaling Technology, Danvers, MA, USA), and anti-β-Actin antibodies (sc-47778, 1:1000 dilution, Santa Cruz Biotechnology, Dallas, TX, USA). The secondary antibody used was HRP-conjugated anti-rabbit (1:5000 dilution; Abmart Inc., Shanghai, China). An imaging system (Amersham Imager 600, GE Healthcare, Chicago, IL, USA) was used for the documentation of the Western blot results. Western blot band intensities were quantified using ImageJ (v1.53, NIH) after converting gels to 8-bit TIFF format. Three technical replicates were analyzed per sample. Uncropped images of Western blots are shown in Figure S3.

### 4.11. Data Analysis

The data analysis was conducted using GraphPad Prism 9.5.1 (GraphPad Software Inc., La Jolla, CA, USA). A one-way ANOVA was used to compare more than two groups, with pair-wise comparisons made using the *t*-test. Survival curves were plotted using the Kaplan–Meier method and analyzed with the log-rank test. *p*-values of <0.05 were considered statistically significant.

## Data Availability

Data are contained within this article.

## Supplementary Materials

The following supporting information can be downloaded at: https://www.mdpi.com/article/10.3390/ijms26178471/s1.
