# Supplementary material for: Neuromedin U Receptor NMUR3 Regulates Autophagy, Thereby Enhancing Thermal Tolerance in C. elegans
Source: Int J Mol Sci. 2025 Aug 31;26(17):8471. doi: 10.3390/ijms26178471 (PMC12428871; doi:10.3390/ijms26178471)
Supplement: Supplementary file 1 [file ijms-26-08471-s001.zip › ijms-3778488-supplementary.pdf]

International Journal of
Molecular Sciences

Supplementary Materials

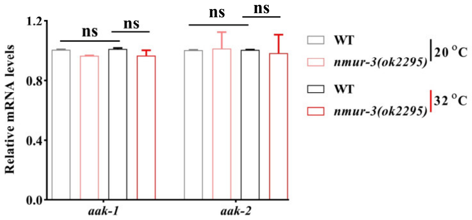

Figure S1. RT-qPCR analysis of *aak-1* and *aak-2* genes expression in wild-type and *nmur-3(ok2295)* mutants at different temperatures. Quantitative RT-qPCR analysis detected unaltered mRNA levels of *aak-1* and *aak-2* in *nmur-3(ok2295)* mutants under normal conditions relative to WT. Quantitative RT-qPCR analysis detected unaltered mRNA levels of *aak-1*and *aak-2* in *nmur-3(ok2295)* mutants following 32 °C heat shock relative to WT. Quantitative RT-qPCR analysis detected unaltered mRNA levels of *aak-1* and *aak-2* in WT worms exposed to 32 °C compared to WT worms exposed to 20 °C. ns, not significant. The results are presented as the means ± SD of three independent experiments. Statistical significance was determined by Student's t-test.

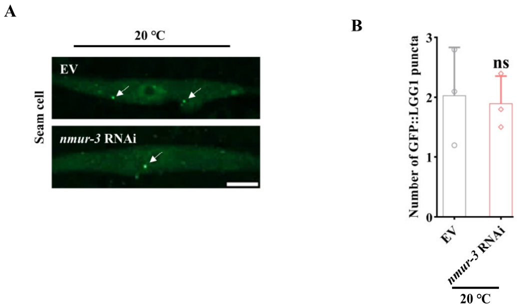

Figure S2. The number of GFP::LGG-1 puncta in the seam cells did not change in *nmur-3* RNAi worms compared to EV worms when they were exposed to 20 °C. (A) Representative images of GFP::LGG-1 puncta in seam cells of worms (scale bar: 2 μm). The arrow indicates a typical GFP::LGG-1 puncta. (B) Quantification of the number of GFP::LGG-1 puncta in seam cells of worms. ns, not significant. The results are presented as the means ± SD of three independent experiments. The *P*-value was calculated using Student's t-test.

Figure 3E

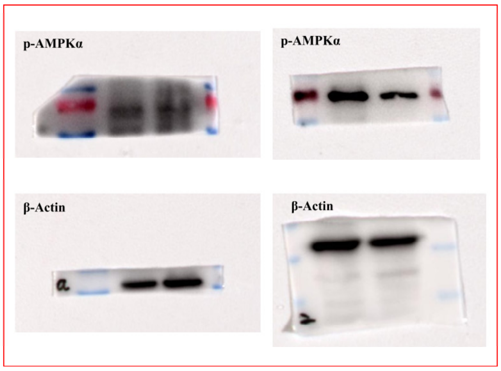

Figure 4A

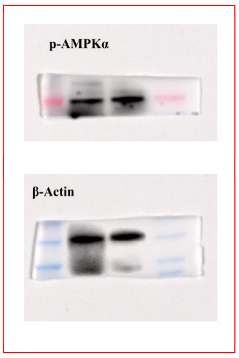

Figure S3. Full scans of the Western blot image.

Table S1. Lifespan and survival analysis of *C. elegans*

**Strain (Genotype)**	**Median survival ± SD (Days)**	**Number of animals**	***P*-value (log-rank test)**	**Figure**
WT	18±1.1	172	--	Figure 1A
nmur-3 (ok2295)	16±2.1	157	0.1576	Figure 1A
WT	8±1	158	--	Figure 1C
nmur-3 (ok2295)	10±0.7	169	0.1150	Figure 1C
**Strain (Genotype)**	**TD Mean ±SD (Hours)**	**Number of animals**	***P*-value (log-rank test)**	**Figure**
WT	68 ± 2.28	80	--	Figure 1B
nmur-3 (ok2295)	70 ± 2.96	85	$P = 0.865$ vs. WT	
WT	40±2.11	55	--	Figure 2B
nmur-3 (ok2295)	28±2.03	51	$P < 0.01$ vs. WT	
EV	48±2.31	52	--	Figure 2C
nmur-3 RNAi	36±1.89	58	$P < 0.01$ vs. EV	
EV	44±2.21	56	--	Figure 4C
nmur-3(ok2295) +EV	30±1.99	52	$P < 0.01$ vs. EV	
aak-2 RNAi	22±2.04	58	$P < 0.01$ vs. EV	
nmur-3(ok2295)+aak-2 RNAi	20±1.96	53	$P = 0.724$ vs. *aak-2* RNAi	
EV	50±1.98	54	--	Figure 5C
nmur-3(ok2295)+EV	38±2.42	58	$P < 0.01$ vs. EV	
bec-1 RNAi	44±2.27	56	$P < 0.01$ vs. EV	
nmur-3(ok2295)+bec-1 RNAi	42±1.64	57	$P = 0.683$ vs. *bec-1* RNAi	
EV	52±2.88	59	--	Figure 5D
aak-2 RNAi	28±2.56	57	$P < 0.01$ vs. EV	
bec-1 RNAi	42±1.98	54	$P < 0.01$ vs. EV	
*aak-2*RNAi+*bec-1* RNAi	26±2.67	56	$P = 0.778$ vs. *aak-2* RNAi	

Table S2. RT-qPCR primers used in this study

Primer name	sequence（5' to 3'）
*sod-3*F	TTCGAAAGGGAATCTAAAAGAAG
sod-3R	GCCAAGTTGGTCCAGAAGATAG
*act-1*F	GCTTCAGTGAGGAGGACTGG
*act-1*R	GTCGGTATGGGACAGAAGGA
*aak-2*F	TGTCGTTGGAAAGATTCGCC
*aak-2*R	CCGTCCGTGCTTAACAATGT
*aak-1*F	TGGAAGAATGAAAGGACTCGG
*aak-1*R	TCGTCGACTTTCTCTGATTGGA
*hsf-1*F	GCATAACAATATGAATAGCATGGTC
*hsf-1*R	GACGTCCTTGTACAAAACACGGATG
*daf-16*F	ATCAGACATCGTTTCCTTCGG
*daf-16*R	TTAACCGTTTCTCTGGACTAGC
*cdc-42*F	TCGACAATTACGCCGTCACA
*cdc-42*R	GAAACACGTCGGTCTGTGGA
*hsp-16.2*F	AGATGTAGATGTTGGTGCAGT
*hsp-16.2*R	TCTCTTCGACGATTGCCTGT
*hsp-70*F	CTACATGCAAAGCGATTGGA
*hsp-70*R	GGCGTAGTCTTGTTCCCTTC
*F44E5.4*F	TGATACCCATCTCGGAGGAG
*F44E5.4*R	GTGGATTGGGTGAAATGTCC
hsp-12.6F	TGGCCACTTCAAAAGGGAG
hsp-12.6R	CTCTTTTGGGAGGAAGTTATGG
